# Integrating personalized medical test contents with XML and XSL-FO

**DOI:** 10.1186/1472-6920-11-8

**Published:** 2011-03-01

**Authors:** Dennis Toddenroth, Martin Dugas, Thomas Frankewitsch

**Affiliations:** 1 IT-Zentrum of the Medical Faculty, University of Münster, Domagkstraße 5, 48149 Münster, Germany; 2 Institute of Medical Informatics, University of Münster, Domagkstraße 9, 48149 Münster, Germany

## Abstract

**Background:**

In 2004 the adoption of a modular curriculum at the medical faculty in Muenster led to the introduction of centralized examinations based on multiple-choice questions (MCQs). We report on how organizational challenges of realizing faculty-wide personalized tests were addressed by implementation of a specialized software module to automatically generate test sheets from individual test registrations and MCQ contents.

**Methods:**

Key steps of the presented method for preparing personalized test sheets are (1) the compilation of relevant item contents and graphical media from a relational database with database queries, (2) the creation of Extensible Markup Language (XML) intermediates, and (3) the transformation into paginated documents.

**Results:**

The software module by use of an open source print formatter consistently produced high-quality test sheets, while the blending of vectorized textual contents and pixel graphics resulted in efficient output file sizes. Concomitantly the module permitted an individual randomization of item sequences to prevent illicit collusion.

**Conclusions:**

The automatic generation of personalized MCQ test sheets is feasible using freely available open source software libraries, and can be efficiently deployed on a faculty-wide scale.

## Background

In 2004 a reshuffle of clinical courses at the medical faculty in Muenster led to the adoption of a modular curriculum and faculty-wide exams. This reorganization aimed to advance the quality of testing and to harmonize requirements among different subjects. Consequently centralized examinations were introduced, predicated on multiple-choice questions (MCQs). When planning the transformation it was taken into account that MCQ-based tests generally allow scores to be calculated in an objective and straightforward fashion. On the other hand this form of testing typically requires an attentive preparation of contents, and is also frequently criticized, for instance for encouraging *'to receive and repeat dogma' *instead of imparting *'an appreciation of method' *[1].

However, realizing large-scale written examinations in conjunction with a modular course system entails several intricate organizational challenges. For example, as students can communicate between successive exam dates, item sets should preferably be used only at a single point in time. Demands to utilize test contents efficiently and to treat candidates equally suggest that items should be presented simultaneously to as many candidates as possible.

A modular curriculum, which allows students to individually sign up to selected courses, contributes to the organizational complexity, because the desired temporal coherence of (personally packaged) tuition and testing implicates that tests should likewise be based on personalized sets of MCQs. Moreover, the need to only sparingly use the time of students and supervisory staff (and to find rooms and test dates) suggests that the number of scheduled test dates should be limited.

These prerequisites were approached by introducing a set of written examinations at the close of every semester. These examinations were structured so that on each test date the contents that are taught in each semester of the programme are encompassed.

To accommodate individual choices regarding test assortments, students were asked to individually sign up for desired test parts, whose partitioning in turn reflected the structure of the related tuition modules. These exam registrations, submitted via a web application, were accumulated in a database together with the designated item contents, and were then used as the data source for the preparation of personalized test sheets.

In this constellation existing packages for electronic testing appear approximately suitable to meet several requirements. For example, open source learning management systems (LMS) such as *Moodle *or *ILIAS*, although primarily geared to online content delivery and course administration, include specific extensions for editing MCQ items and for holding quizzes. (These platforms are also comparable in their technical approach, because users access both via a web browser, and because for storage both rely on relational databases [2,3].)

In our setting the technical infrastructure for electronic testing was not available at the time, so it was decided to initially introduce examinations based on personalized printouts. Such an approach, which may seem out-dated in comparison with electronic testing, can also result in several specific advantages; for example, paper-based tests are intuitively usable and directly yield an indelible documentation that can be filed away.

In principle personalized exam sheets could be generated by scripting a word processor, or alternatively just by using paper and scissors. However, the scale of the projected processing needs of hundreds of individually personalized tests per semester prompted automating the task by implementing a specialized software module.

As the procedure already requires a reproducible personalization of item sets, an individual randomization of item sequences becomes easily attainable. This may help to impede collaboration between students in an examination environment, as it may prevent potential cheaters from relying upon the sequence of test items to identify equal contents and copy answers.

Below we therefore report on key technical aspects of the module (demonstrated by the software supplement in 'additional file 1'), as well as on experiences collected during five years of operation.

Note that in our setting authors and administrative staff had accessed a different module for the collaborative entry and revision of items. This component is technically more comparable to the mentioned LMS platforms (which also store these contents in the tables of a relational database), but is at the same time more closely adapted to local administrative settings. The following technical description therefore focuses on the module for sheet assembly.

## Implementation

At the outset of the procedure, MCQ contents and individual test registrations are accumulated in a single database. The described method for generating personalized examinations involves querying relevant subsets of item contents and test registrations, integrating these into personalized Extensible Markup Language (XML) documents, and sequentially transforming these into PDF files for printing. After a characterization of the required input data, these key steps are discussed in more detail below.

### Specifications of input data

The software package for the generation of personalized test sheets has to account for some additional practical facets. The need for emphasis of item parts, such as crucial keywords or proper names, calls for basic formatting capabilities (e.g. boldface or italics), which should support recombination. Such formatting elements aim to enhance test accessibility, and might avoid irrelevant difficulty that might detract from the intended competency measurements [4].

To store formatting directives in the database, textual MCQ contents are encoded by using the "flow content" markup elements from transitional *Extensible HyperText Markup Language *(XHTML) [5], i.e. those elements that are commonly related to physical text appearance.

MCQ authors also frequently use characters outside the regular ASCII character set. Diacritical marks, such as German umlaute, French accents, and Greek letters are in regular use, for example to denote pharmacological targets. A predefined set of XHTML entities are used for non-ASCII characters, so that the formatted phrase *"...a temperature of **38,9°C**" *would in the database be encoded as *"...a temperature of* *<u> <b> 38,9&deg;C </b> </u>"*. (Note that showing text simultaneously bold and underlined is achieved by nesting the corresponding markup elements.)

Assessing the competency to interpret medical images motivates using graphics in MCQ items, which may often contain photographs of pathology, radiologic imaging, or abstract diagrams such as statistical plots. These graphical media, which in the database are stored as Binary Large Objects (BLOBs), should in printouts ideally appear next to the textual contents, as this dispenses with the need for consulting a separate image supplement.

All in all these formatting elements, extended character sets, and included graphical media aim to increase the realism of the exam, since such facets are also frequently encountered in original medical documentation.

### Querying relevant item contents

**Figure 1:**
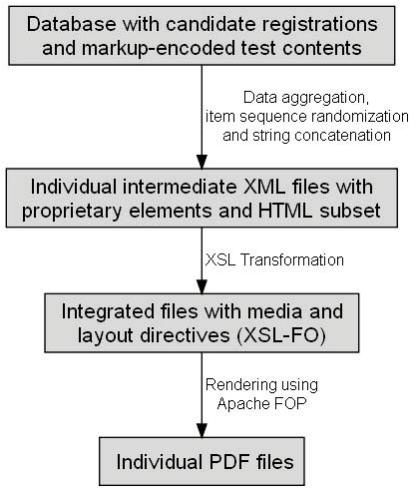
**Data flow**. This diagram illustrates the key steps of the preparation of tailored test sheets from a database of impersonal MCQ items and individual test registrations.

The data flow diagram in figure 1 provides an overview of the main stages of the procedure. After specifying basic parameters for the selected examination, all necessary information is retrieved with *Structured Query Language *(SQL) commands. Querying relevant data occurs in a biphasic fashion. Initially students that registered for the particular test date are identified, before individually needed contents are collected during a subsequent iteration over these registered students.

During this stage individual pseudo-randomized item sequences are assigned, coded in SQL as an ORDER BY directive. These item sequences are concomitantly inserted into another table of the database, so that after the examination the mapping between individual answers and MCQ contents can be reproduced.

### Assembling XML documents

All data that constitute a personalized test are then concatenated into a single temporary XML document. Within these intermediates a preconceived set of markup elements establishes a simple hierarchical structure. For example, the root element embodies a personalized test sheet as the overriding ancestor, while its descendants represent single MCQ items. Referenced graphical media are automatically embedded into these XML files using Base64 encoding.

The XML document is not formally validated (e.g. using a DTD or XML Schema), because the program logic ensures that only meaningful intermediates are automatically generated. Consecutive parsing and transformation is already operable on well-formedness of the document.

### Transformation into PDF files with an open source print formatter

At this stage test sheet representations are already integrated into single files, and comply with a particular syntactical structure, but still need directives to control layout and pagination.

These presentational instructions are attached during the ensuing conversion into *Extensible Stylesheet Language - Formatting Objects *(XSL-FO), itself a markup-based language that serves as a precursor of page description languages. Conversion operations are defined as XML-encoded templates in *XSL Transformation *(XSLT) stylesheets. These complementing technologies were collectively formalized by the World Wide Web Consortium (W3C) as *Extensible Stylesheet Language *[6].

XSL is thereby employed for setting parameters such as page size and margins, for counting and enumerating elements, and for assembling a preamble page that summarizes item frequencies. To achieve an intuitively accessible appearance of test contents, layout directives were chosen so that for example page breaks should not disrupt the consecutive answer options of a common MCQ.

XHTML-coded formatting directives and entities are intercepted and converted into suitable XSL-FO elements and special characters. A specific element (termed *external-graphic*) within XSL-FO is used to display enclosed images.

These self-contained XSL-FO documents are consecutively rendered into Portable Document Format (PDF) files by invoking Apache FOP. This freely available open source print formatter from the Apache XML Graphics Project [7] automatically applies formatting directives, and sets line breaks and page breaks. Output PDFs are then gathered in the file system before printing.

The presented procedure can be more comprehensively automated. In our setting a tailored Java program attends to all required steps, which may be controlled via a minimalist graphical user interface (GUI) based on the *Standard Widget Toolkit *[8]. (Such further automation is not contained in the supplemented software demonstration, as we conjecture it will often require a close-mesh customization to organizational specifics such as a particular curricular structure.)

## Results

So far the described software module was employed for five years of continuous operation between 2005 and 2010. During this time more than 12.000 personalized tests were generated in preparation for more than 60 single examination dates. On each test date personalized sheets were assembled for approximately 160 candidates. Over time some 2000 individual students participated. Most candidates attended several test dates as they progressed with their studies.

Collectively almost 13.000 discriminate MCQ items were employed, related to more than 30 different curricular subjects. These contents were disposed so that the whole set of all personalized sheets contained roughly 1.5 million singular item printouts, corresponding to the expected total of individual responses during these tests (disregarding that in practice a minority of these items remained unanswered).

**Figure 2:**
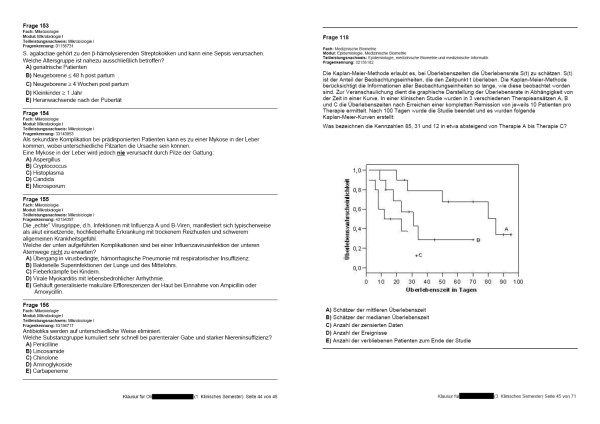
**Sample outputs**. Images of test sheets from automatically created personalized exams, illustrating exemplary page layout results. Real sheet size conforms to German DIN standard 476 (ISO 216; 210 mm × 298 mm). XSL-FO directives configure that candidate names (blackened) and page numbers are automatically integrated into every page footer. Modes of numerating MCQ items, answer choices, and pages are defined within XSL stylesheets. All numbers are automatically assigned during the XSL transformation. Formatting characteristics, such as font sizes or margins between paragraphs, are also declared within the XSL stylesheets.

The rendering library consistently produced high-quality PDF outputs. Line breaks and page breaks were dependably inserted in reasonable positions, leading to clearly accessible paragraphs (figure 2 shows two samples pages).

Since both vectorized elements (textual contents) and pixel graphics were integrated into PDF outputs, observed file sizes were quite efficient. A typical personalized test, which in our setting often consisted of approximately 180 one-out-of-five MCQ items, may have been stored as a PDF file between several hundred kilobytes and a few megabytes, strongly depending on the quantity and resolution of embedded graphics.

As a consequence of the structured design of the module, with styling definitions for sheet outputs separated from the residual program logic, a relatively straightforward adaptability was attained. For example, the software could be rapidly adjusted to changes of the underlying database structure with modifications just to the set of SQL commands.

Likewise, the functionality of the software could be gradually extended. Available formatting options were by and by upgraded beyond elementary features (namely boldface, underline or italics), so that later versions also supported tabulations, enumerations or unnumbered itemizations, and allowed rendering text as superscript or subscript.

## Discussion

We reported on the design of a made-to-measure software module for the automatic preparation of personalized medical test sheets, predicated on a database of individual test registrations and MCQ contents. During five years of operation the system consistently produced high-quality PDF outputs.

This practical experience demonstrates that the technical approach, in terms of the scalability of key parameters such as item frequencies and candidate numbers, is applicable in a faculty-wide setting. However, the observed performance does not imply any fixed technical constraint; the approach may also be capable of handling more extensive datasets (i.e. that it might also be deployed if individual tests consisted of more MCQ items, or if more candidates were attending each test date).

As the software module was entirely assembled from freely available open source components, no costs for software licenses were incurred. It may seem noteworthy that, although these technologies were primarily designed and standardized for the World Wide Web, the application does not specifically rely on usage in conjunction with a computer network.

Today XML is widely used in medical documentation and biological research. This acceptance may be attributable to the fact that it is specifically geared towards automated processing and yet more flexible than for example hypertext [9]. XSLT, which provides a standardized method of transforming and converting XML documents, is mentioned less frequently in these contexts.

Reportedly XSLT-based procedures were also successfully applied in specific areas of clinical documentation [10,11]. Our software module may thus be seen as a comparable example of how XSLT can be used to automate the conversion of structured medical data into human readable documents.

Currently we plan to extend the functionality to permit inclusion and processing of other types of data into MCQ items (e.g. mathematical formulae), and to explore interfaces for electronic testing. Moodle and ILIAS, for example, both implement electronic quizzes by presenting contents as HTML input forms that can be worked on in a web browser. As different XSL stylesheets could be specified to generate HTML syntax instead of XSL-FO, personalized XML intermediates appear to constitute a reasonable basis to produce such an alternative output target. (The software supplement in 'additional file 1' also contains an alternative stylesheet that creates statically prearranged HTML outputs to demonstrate a basic compatibility with browser-based methods of holding electronic quizzes.)

Conversely the underlying (SQL-based) databases of these learning management systems might also come into consideration as data sources to drive the described PDF-generation. For example, Moodle already allows exporting MCQ contents as (impersonal) XML files, which could in principle also be transformed into XSL-FO. The stylesheets would have to be moderately adjusted to reflect the particular composition of elements and attributes within these intermediates. However, a comparable personalization of test sheets would also require that individual item sequences remain traceable, for example by defining another database table as demonstrated in the supplement.

According to circumstantial recounts, the introduction of individually randomized item sequences was accompanied by an improved quiescence and attentiveness during the examination. This supports our assumption that such a technique can prevent cheating, and can consequently assist in administering fairer, more reliable, and thus more valid test results. According to [12], preventing collusion would belong to reinforcing the 'response process' category of validity criteria, as it aims to hold candidates off sidestepping designated 'thought processes'.

Whitley in an overview of the determinants of cheating concluded that using more than one test form with permuted item sequences has a 'slight inhibitory effect' - less effective, for instance, than having candidates seated with an empty chair between adjacent students rather than immediately next to each other [13]. These measures could obviously also be applied simultaneously.

Our presented approach of also randomizing item sequences on an individual basis could enhance the preventive effectiveness on cheating, because it may in equal measure inhibit collusion between any given pair of candidates, while preparing only two test versions might reportedly prompt cheaters to resort to copying from candidates seated in front of them [14].

In the future assessing such presumed preventive effects could involve calculating statistical indices to detect elevated response similarities between communicating candidates as discussed by Angoff [15]. The vast quantity of answer choices for that purpose might then require an automated computation of these similarity measures, a course of action demonstrated by McManus et al. [16].

Houston suggests that also randomizing the sequence of answer options might have additional preventive effects on cheating [17]. Such a strategy might constitute another useful extension of the presented software module, although it would necessitate additional data storage for also preserving permutations of individual sequences of answer options.

## Conclusions

Automating the procedure for generating personalized test sheets from a database of MCQ contents and individual test registrations is feasible and efficient on a faculty-wide scale. Further research should investigate whether (or how much) such individually randomized item sequences impede illicit collusion during the examination.

## Availability and requirements

Project name: none

Project home page: none (an open-source demonstration is included in 'additional file 1')

Operating system(s): Platform-independent

Programming language: XSLT, SQL, Java/Python

Other requirements: Apache FOP, a database with test contents

License: Apache 2.0

Any restrictions to use by non-academics: none

## Competing interests

The authors declare that they have no competing interests.

## Authors' contributions

All authors participated in the coordination and design of this study, contributed to the interpretation of results, revised the manuscript, and read and approved the final paper. DT conceived and implemented the described software module in its original form and drafted this manuscript. TF operated the software and extended its functionality.

## Pre-publication history

The pre-publication history for this paper can be accessed here:

http://www.biomedcentral.com/1472-6920/11/8/prepub

## Supplementary Material

Additional file 1 **This archive contains files that demonstrate key technical concepts of the described software module**. Inputs can be found in a subfolder 'source' (including XSL stylesheets), while 'output' contains sample results (including XML intermediates). A 'README' file in the root folder provides additional information and a short recipe. Click here for file
