# Supplementary material for: Integrating personalized medical test contents with XML and XSL-FO
Source: BMC Med Educ. 2011 Mar 1;11:8. doi: 10.1186/1472-6920-11-8 (PMC3056837; doi:10.1186/1472-6920-11-8)
Supplement: Additional file 1 — This archive contains files that demonstrate key technical concepts of the described software module. Inputs can be found in a subfolder 'source' (including XSL stylesheets), while 'output' contains sample results (including XML intermediates). A 'README' file in the root folder provides additional information and a short recipe. [file 1472-6920-11-8-S1.ZIP › output/pdf/exam_1.pdf]

Jane Doe

This exam consists of 2 items:

Curricular context	Items
Cytogenetics	2

Item 1 (Cytogenetics)

Which of the given karyotypes is most likely associated with the following set of symptoms?
- omphalocele
- esophageal atresia
- micrognathism
- clenched hands
- overlapping fingers

A) 47,XX,+21

B) 47,XXY

C) 47,XX,+16

D) 45,X

E) 47,XX,+18

Item 2 (Cytogenetics)

Which of the human chromosomes is shown?

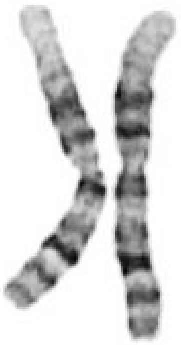

A) 1

B) 5

C) 9

D) 16

E) 21
