# Supplementary material for: Integrating personalized medical test contents with XML and XSL-FO
Source: BMC Med Educ. 2011 Mar 1;11:8. doi: 10.1186/1472-6920-11-8 (PMC3056837; doi:10.1186/1472-6920-11-8)
Supplement: Additional file 1 — This archive contains files that demonstrate key technical concepts of the described software module. Inputs can be found in a subfolder 'source' (including XSL stylesheets), while 'output' contains sample results (including XML intermediates). A 'README' file in the root folder provides additional information and a short recipe. [file 1472-6920-11-8-S1.ZIP › output/pdf/exam_2.pdf]

John Doe

This exam consists of 4 items:

Curricular context	Items
Cytogenetics	2
Pharmacology	2

Item 1 (Cytogenetics)

Which of the given karyotypes is most likely associated with the following set of symptoms?
• omphalocele
• esophageal atresia
• micrognathism
• clenched hands
• overlapping fingers

A) 47,XX,+21

B) 47,XXY

C) 47,XX,+16

D) 45,X

E) 47,XX,+18

Item 2 (Pharmacology)

What should be measured to control and adjust the dosage of oral synthetic thyroid hormones after thyroidectomy?

A) Plasma concentration of thyrotropin-releasing hormone (TRH)

B) Plasma concentration of thyroid-stimulating hormone (TSH)

C) Plasma concentration of triiodothyronine (T_3)

D) Plasma concentration of thyroxine (T_4)

E) Body temperature and heart rate

Item 3 (Pharmacology)

What is a suitable therapy for **acute asthmatic bronchoconstriction**?

A) Tamsulosin (α_1 receptor antagonist)

B) Clonidine (α_2 receptor agonist)

C) Dobutamine (β_1 receptor agonist)

D) Salbutamol (β_2 receptor agonist)

E) Atenolol (β_1 receptor antagonist)

Item 4 (Cytogenetics)

Which of the human chromosomes is shown?

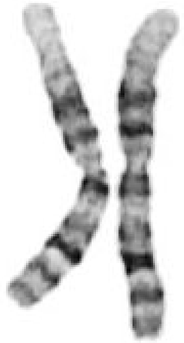

A) 1
B) 5
C) 9
D) 16
E) 21
