# Supplementary material for: Integrating personalized medical test contents with XML and XSL-FO
Source: BMC Med Educ. 2011 Mar 1;11:8. doi: 10.1186/1472-6920-11-8 (PMC3056837; doi:10.1186/1472-6920-11-8)
Supplement: Additional file 1 — This archive contains files that demonstrate key technical concepts of the described software module. Inputs can be found in a subfolder 'source' (including XSL stylesheets), while 'output' contains sample results (including XML intermediates). A 'README' file in the root folder provides additional information and a short recipe. [file 1472-6920-11-8-S1.ZIP › output/xhtml/exam_2.html]

xml version="1.0" encoding="ISO-8859-1"?


John Doe


## John Doe

---

This exam consists of 4 items:

| Curricular context | Items |
| --- | --- |
| Cytogenetics | 2 |
| Pharmacology | 2 |

---


Item 1
(Cytogenetics)

Which of the given karyotypes is most likely associated with the following set of symptoms? 

- omphalocele
- esophageal atresia
- micrognathism
- clenched hands
- overlapping fingers

|  |  |
| --- | --- |
|  | A) 47,XX,+21 |

|  |  |
| --- | --- |
|  | B) 47,XXY |

|  |  |
| --- | --- |
|  | C) 47,XX,+16 |

|  |  |
| --- | --- |
|  | D) 45,X |

|  |  |
| --- | --- |
|  | E) 47,XX,+18 |

---

Item 2
(Pharmacology)

What should be measured to control and adjust the dosage of oral synthetic thyroid hormones after thyroidectomy?

|  |  |
| --- | --- |
|  | A) Plasma concentration of thyrotropin-releasing hormone (TRH) |

|  |  |
| --- | --- |
|  | B) Plasma concentration of thyroid-stimulating hormone (TSH) |

|  |  |
| --- | --- |
|  | C) Plasma concentration of triiodothyronine (T3) |

|  |  |
| --- | --- |
|  | D) Plasma concentration of thyroxine (T4) |

|  |  |
| --- | --- |
|  | E) Body temperature and heart rate |

---

Item 3
(Pharmacology)

What is a suitable therapy for acute asthmatic bronchoconstriction?

|  |  |
| --- | --- |
|  | A) Tamsulosin (α1 receptor antagonist) |

|  |  |
| --- | --- |
|  | B) Clonidine (α2 receptor agonist) |

|  |  |
| --- | --- |
|  | C) Dobutamine (β1 receptor agonist) |

|  |  |
| --- | --- |
|  | D) Salbutamol (β2 receptor agonist) |

|  |  |
| --- | --- |
|  | E) Atenolol (β1 receptor antagonist) |

---

Item 4
(Cytogenetics)

Which of the human chromosomes is shown?

|  |  |
| --- | --- |
|  | A) 1 |

|  |  |
| --- | --- |
|  | B) 5 |

|  |  |
| --- | --- |
|  | C) 9 |

|  |  |
| --- | --- |
|  | D) 16 |

|  |  |
| --- | --- |
|  | E) 21 |

---
